# Postnatal development of the relaxin-3 innervation of the rat medial septum

**DOI:** 10.3389/fnins.2023.1176587

**Published:** 2023-05-10

**Authors:** Francisco Ros-Bernal, Isis Gil-Miravet, Jorge Lucerón, Mónica Navarro-Sánchez, Esther Castillo-Gómez, Andrew L. Gundlach, Francisco E. Olucha-Bordonau

**Affiliations:** ^1^Unitat Predepartamental de Medicina, Facultad de Ciencias de la Slud, Universitat Jaume I, Castellón, Spain; ^2^Centro de Investigación Biomédica en Red de Salud Mental, (CIBERSAM), Madrid, Spain; ^3^The Florey Institute of Neuroscience and Mental Health, Florey Department of Neuroscience and Mental Health and Department of Anatomy and Physiology, The University of Melbourne, Parkville, VIC, Australia

**Keywords:** diagonal band, neuropeptide, nucleus incertus, RXFP3, septohippocampal system, serotonin, theta rhythm

## Abstract

**Introduction:**

The septal area provides a rich innervation to the hippocampus regulating hippocampal excitability to different behavioral states and modulating theta rhythmogenesis. However, little is known about the neurodevelopmental consequences of its alterations during postnatal development. The activity of the septohippocampal system is driven and/or modulated by ascending inputs, including those arising from the nucleus incertus (NI), many of which contain the neuropeptide, relaxin-3 (RLN3).

**Methods:**

We examined at the molecular and cellular level the ontogeny of RLN3 innervation of the septal area in postnatal rat brains.

**Results:**

Up until P13–15 there were only scattered fibers in the septal area, but a dense plexus had appeared by P17 that was extended and consolidated throughout the septal complex by P20. There was a decrease in the level of colocalization of RLN3 and synaptophysin between P15 and P20 that was reversed between P20 and adulthood. Biotinylated 3-kD dextran amine injections into the septum, revealed retrograde labeling present in the brainstem at P10-P13, but a decrease in anterograde fibers in the NI between P10–20. Simultaneously, a differentiation process began during P10–17, resulting in fewer NI neurons double-labeled for serotonin and RLN3.

**Discussion:**

The onset of the RLN3 innervation of the septum complex between P17–20 is correlated with the onset of hippocampal theta rhythm and several learning processes associated with hippocampal function. Together, these data highlight the relevance and need for further analysis of this stage for normal and pathological septohippocampal development.

## 1. Introduction

The complex development of the central nervous system (CNS) is the result of two main biological processes, the first being an intricate genetically-encoded program controlling cell division and differentiation, and the second, the mutual interaction between the emerging neural and non-neuronal components and external environmental factors. The classical model of development describes two phases. The initial phase of predetermined genetic programs produces the general pattern and divisions of the CNS and a coarse network of connections between the main brain divisions (Rubenstein et al., 1994, 1998; Martínez and Puelles, 2000; Puelles et al., 2000). During the final phase, the neural connections are refined as a result of interactions between the nervous system and the outside world (Blankenship and Feller, 2010). However, this view is being refined in light of new observations of interactions between the genetic program and neural activity throughout development to determine the components and organization of developing neural circuits (Ben-Ari et al., 1989; Spitzer, 2006).

The septal area derives from the medial telencephalic wall and is composed of cells of both pallial and subpallial origin (Puelles et al., 2000). Lhx5 is a specific marker for the septal region in the E13.5 stage murine brain. In contrast, this region does not express other genes such as Lhx6, Lhx7 or Couptf1, which are specific for the ganglionic subpallial eminences (Flames et al., 2007). Notably, cholinergic neurons of the basal forebrain arise from a dual origin – pallial cells of the rostromedial telencephalon migrate ventrally following a ventromedial route to populate the diagonal band of Broca and the olfactory tubercle (García-Moreno et al., 2007); while cells positive for reelin and expressing Tbr-1 migrate from the ventral pallium to the subpallium and differentiate into cholinergic neurons in the basal forebrain nuclei projecting to the cortex (Pombero et al., 2011). Medial septal and vertical diagonal band neurons derive from the subpallial medial ganglionic eminence cells expressing the homeodomain transcription factor, Nkx2.1 (Xu et al., 2008).

The neurogenesis of cholinergic basal forebrain neurons in rats occurs during embryonic (E) day 12 to E16 and they are among the first cells to leave the mitotic cycle (Semba and Fibiger, 1988; Brady et al., 1989). Retrograde tracing studies reveal that the basal forebrain cholinergic projection to the visual cortex is already remarkeable remarkably mature at P0 (de Carlos et al., 1995). However, cholinergic neurons in the basal forebrain increase in number, size, and complexity until the end of the third postnatal week (Gould et al., 1991).

BrdU experiments reveal that septal neurons are born mainly between E10.5 and E14 in mice (Wei et al., 2012). Once generated, the cells initiate a process of radial, tangential, or local migration towards their final location, which generates the resulting onion-shaped septal structure (Wei et al., 2012). Throughout this time and until the end of the third postnatal week, septal neurons undergo a maturation process involving phases of different marker gene/protein expression.

Four groups of nuclei can be identified according to their anatomical location, in the medial basal region. The medial group consists of the medial septal complex, which includes the medial septal nucleus (MS) and the nucleus of the diagonal band, divided into its horizontal (HDB) and vertical (VDB) arms. The adjacent lateral group is composed of the lateral septal, septofimbrial and septohippocampal nuclei. The ventral group comprises the nuclei of the bed of the stria terminalis (BST) and lastly, the posterior group consists of the bed nuclei of the anterior commissure and the stria medullaris, and the triangular nucleus (Zaborszky et al., 2015).

In the adult rat, the MS receives ascending projections from the posterior hypothalamus, including the supramammillary nuclei (Vertes, 1992; Leranth and Kiss, 1996; Kiss et al., 2000; Vertes and McKenna, 2000), brainstem raphe nuclei (Kocsis and Vertes, 1992; Vertes et al., 1994; Vertes and Kocsis, 1994; Vertes and McKenna, 2000; McKenna and Vertes, 2001) and the NI in the pontine tegmentum (Goto et al., 2001; Olucha-Bordonau et al., 2003). The characterization of these ascending connections from the hypothalamus and brainstem to the septum and hippocampus led to the classical view of the MS as the main modulator or driver of hippocampal theta rhythm (Vertes and Kocsis, 1997). In addition, the MS sends descending projections to the supramammillary nuclei (Borhegyi and Freund, 1998; Leranth et al., 1999) and the NI (Sánchez-Pérez et al., 2015), although these are less abundant than the ascending projections. Nonetheless, bidirectional connections between the hippocampus and the NI that pass through the MS result in coherence waves within these structures (Martínez-Bellver et al., 2017).

Neurons of the rat NI express the neuropeptide, relaxin-3 (RLN3) (Burazin et al., 2002; Tanaka et al., 2005). RLN3 is expressed largely within GABAergic neurons (Ma et al., 2007), but there are some RLN3-positive NI neurons that express the glutamate vesicular transporter 2 (vGlut2), indicative of a possible excitatory phenotype (Szlaga et al., 2022). A G_i/o_-protein-coupled receptor, RXFP3 (formerly called GPCR135), is the cognate receptor for RLN3 (Liu et al., 2003; Chen et al., 2005) and is highly expressed in rat brain areas targeted by RLN3 fibers arising from the NI (Sutton et al., 2004; Ma et al., 2007). The NI projections to the MS have been directly associated with the modulation of hippocampal theta rhythm (Nuñez et al., 2006). Stimulation of the NI increased the theta band in the CA1 field potential in urethane-anaesthetized rats (Nuñez et al., 2006). Moreover, the same increase in hippocampal theta rhythm was obtained after the intra-septal infusion of an RXFP3 agonist, and was prevented by the prior infusion of an RXFP3 antagonist (Ma et al., 2009). In addition, intracerebroventricular (icv) infusion of an RXFP3 agonist resulted in increased ERK phosphorylation in cholinergic neurons, and disruption of spatial working memory performance in the spontaneous alternation task (Albert-Gascó et al., 2017). Furthermore, depletion of RXFP3 expression in the MS of floxed-Rxfp3 mice resulted in disruption of spatial memory in the Morris water maze (Haidar et al., 2019).

Until the current studies, there had only been a small number of reports on the development of the brain RLN3-RXFP3 system. However, it has been documented that RXFP3 is expressed in brain before birth, but RLN3-positive neurons first appear after birth and are then fully established by P7 (Miyamoto et al., 2008). NI neurons express the serotonin (5HT) 5HT_1A_ receptor and transient postnatal depletion of 5HT resulted in an increase in RLN3 expression in NI neurons (Miyamoto et al., 2008). On this basis, a postnatal regulation of RLN3 levels in the NI by 5HT was postulated.

We have demonstrated that the NI impacts theta-related hippocampal function by targeting different neuronal types in the MS that are directly involved in the generation and modulation of theta rhythm (Olucha-Bordonau et al., 2012). Therefore, we hypothesized that the development of the RLN3 innervation of the septal area is related to the maturation of specific neuronal types and the neural functions regulated by this system. Thus, in the current studies, we analyzed septal RXFP3 expression levels, the appearance of RLN3 immunoreactive nerve fibers, the development of synaptic contacts in the septal area, and the nature of the 5HT-RLN3 segregation in the NI, at different postnatal stages of rat brain development.

## 2. Materials and methods

### 2.1. Animals

Pregnant Wistar rats were obtained from the Experimental Animal Center of the University Jaume I, Castellón. The day of birth was designated as the first postnatal day (P1) for the offspring. The rats were treated in accordance with the European Community Council Directive (EEC/86/609; 2010/63/EU), Spanish directive BOE 34/11370/2013, and local directive DOGV 26/2010. The Ethics Committee of the University Jaume I approved all protocols, and all efforts were made to minimize the number of rats used and any potential pain or distress.

### 2.2. RT-PCR protocol

Rat pups were killed with an overdose of isoflurane, and brains (n = 4 per postnatal day) were immediately removed and immersed in liquid nitrogen, and then dissected into specific regions including the septal area, and immediately stored at −80°C until RNA preparation. Rats at early postnatal stages were used – days 3 (P3), 10 (P10) and 20 (P20). Septal RXFP3 mRNA levels in these rats were compared with those detected in mature adult rats (9 months-old). Total RNA was prepared from samples using QIAZOL lysis reagent (RNeasy Lipid Tissue Mini Kit, Qiagen, Maryland, USA), as per the manufacturer’s procedures. The concentration of RNA was estimated by spectrophotometry using absorbance at 260 nm. Each 1 μg of RNA were submitted to one-step RT-PCR analysis using a PrimeScript RT Reagent Kit (Takara Bio USA Inc., San Jose, CA, USA). The housekeeping gene, GADPH, was used as an internal control. The PCR primer sequences were as follows: RXFP3 sense primer, 5′-TCGGTGACCATCGTAGTCCT-3′, reverse primer, 5′-CAGCCCGTGATCTTCAGGTT-3′; GADPH sense primer, 5′-TCCACCACCCTGTTGCTGTA-3′, reverse primer, 5′-ACCACAGTC-CATGCCATCAC-3′. Thermal cycling conditions consisted of 40 cycles of 10 s at 95°C, 10 s at 60°C, and 20 s at 72°C. RXFP3 mRNA levels were subsequently normalized to GAPDH mRNA levels.

### 2.3. Surgical procedures

Rats reared in the laboratory until the age of P8, P15 and P18 were used. All rats were carefully separated from their mother for 3 h and placed in an incubator to prevent heat loss. Each rat was anesthetized with isoflurane (Isoflutek, 1 g/g Karizoo Laboratories, Barcelona, Spain) and placed in a Kopf stereotaxic apparatus for surgery. During surgery, rats were mechanically ventilated with 1.5% isoflurane anesthesia (Isoflurane Vaporizer, VetEquip Inc., Pleasanton, CA, USA) in 100% oxygen, and were maintained at 37.5°C with a feedback-controlled heating pad (Bedsure, New York, NY, USA). We used a 22-gauge needle to make a small hole in the skull bone and a 1 μL Hamilton syringe was used for intracranial infusions of 3-kD biotinylated dextran amines (BDA) into the MS. Coordinates were modified for each age: for P8 AP: +0.3; ML: –0.2; DV: −5 mm; for P15, AP: +0.5; ML: –0.2; DV: −5.5 mm; for P18, AP: +0.5; ML: –0.2; DV: −5.5 mm. The infusion volume of BDA, the dual anterograde and retrograde tracer, was 0.3 μL and was delivered at an infusion rate of 0.1 μL/min and a 5 min diffusion time. After the infusion, the skin was sutured and all rats received analgesic treatment with meloxicam (Metacam, 2 mg/kg, 5 mg/ml, Boehringer-Ingelheim, Barcelona, Spain). Following recovery to wakefulness, all rats were returned to their cages and allowed to recover for 2 days prior to further processing.

### 2.4. Brain tissue preparation

Rats were deeply anesthetized with an intramuscular injection of sodium pentobarbital (Dolethal, 200 mg/kg i.p; Vetoquinol S.A., Madrid, Spain) and transcardially-perfused with a solution of NaCl 0.9% and heparin (50 ml) followed by an ice-cold fixative (~50 ml of 4% paraformaldehyde in 0.1 M PB, pH 7.4). Brains were dissected and immersed in the same fixative for 24 h at 4°C and equilibrated in 30% sucrose in 0.01 M PBS pH 7.4 for 48 h at 4°C. Coronal sections (40 μm) were collected using a freezing-slide microtome (Leica SM2010R, Leica Microsystems, Heidelberg, Germany). For each brain, six series of sections were obtained and collected free-floating in cryoprotectant solution (30% ethylene glycol, 30% glycerol in 0.1 M PBS) at −20°C.

### 2.5. Immunohistochemistry for RLN3

The distribution of RLN3 immunoreactive fibers was assessed using immunohistochemistry with a monoclonal RLN3 antiserum raised against the A-chain of the native RLN3 peptide as described (Tanaka et al., 2005; Ma et al., 2013). The specificity of the antiserum has been demonstrated using brain sections from relaxin-3 gene knockout mice (Watanabe et al., 2011). Stocks of the antiserum were produced in-house at The Florey Institute for Neuroscience and Mental Health (Parkville, Australia) and the University Jaume I (Castellon, Spain).

Brain sections (20 μm) from P3, P10 and P20 rats were placed into a Tris buffer +0.2% gelatin solution for 1 min and then incubated at 37°C. After 2 h, sections were washed three times in PBS and treated for 15 min in 0.3% hydrogen peroxide, washed three times in PBS-Tx (PBS containing 0.3% Triton X-100), and incubated in 2% bovine serum albumin and 4% normal goat serum (containing 0.3% Triton X-100 in PBS) for 1 h at room temperature, prior to overnight incubation with a 1:5 dilution of mouse anti-RLN3 (culture media) at room temperature. After a 3 × 10 min wash in PBS-Tx, sections were incubated in biotinylated secondary antibody (goat anti-mouse IgG; Sigma-Aldrich, St Louis, MO, USA; 1:200) at room temperature for 2 h. The sections were then rinsed 3 × 6 min in PBS-Tx and incubated in avidin-biotin peroxidase complex (ABC kit, Sigma; 1:200) for 2 h at room temperature. Following thorough rinsing with Tris-buffer solution (pH 8, 2 × 6 min), immunolabeling was revealed as a black reaction product by immersing the sections in 0.025% 3,3′-diaminobenzidine (DAB; Sigma-Aldrich, St Louis, MO, USA), 0.0024% H_2_O_2_ in Tris HCl, pH 8.0. After staining, sections were dehydrated, cleared and coverslipped.

### 2.6. Immunohistochemistry for RLN3 and calcium-binding proteins

The distribution of RLN3 immunoreactive fibers in relation to the distribution of neurons expressing calcium-binding proteins (CBP), specific neurochemical/anatomical markers, was assessed using immunohistochemistry. An average of six rats per group (P10, P13, P15, P17, P20 and adult) was used. For each rat, three sections from multiple rostrocaudal levels were collected covering the entire medial septal region (0.70 to 0.20 mm, relative to Bregma).

For double RLN3-CBP immunofluorescence, sections were rinsed three times in 0.01 M PBS and transferred to blocking solution (10% normal goat serum (NGS), 2% BSA and 0.1% Triton X100 in PBS) for 1 h at room temperature. Sections were then transferred to primary antibody solution containing mouse anti-RLN3 (1:5, cell culture media) and a single anti-CBP antibody. Four different anti-CBP antibodies were used: rabbit anti-parvalbumin (1:5000, Swant, Burgdorf, Switzerland), rabbit anti-calbindin (1,5,000, Swant, Burgdorf, Switzerland), rabbit anti-ChAT (1,1,000, ThermoFisher Scientific; Valencia, Spain) and rabbit anti-calretinin (1,2000, Swant; Burgdorf, Switzerland). Primary antibodies incubations were performed overnight at room temperature. Sections were rinsed twice in PBS and incubated in 1:200 goat anti-mouse-Alexa 488 antibody (#115–545-146, Jackson ImmunoResearch; Cambridge, United Kingdom) and 1:200 goat anti-rabbit-Cy3 antibody (#111–166-045, Jackson ImmunoResearch; Cambridge, United Kingdom) for 2 h at room temperature. Finally, sections were rinsed in PBS, mounted on clean slides and coverslipped with Fluorsave™ (345,789, Merck Life Science S.L.U., Madrid, Spain).

### 2.7. Immunohistochemistry for RLN3 and synaptophysin

A quantitative assessment of the level of colocalization of synaptophysin (Syn) within RLN3 fibers was conducted using sections double-reacted with the monoclonal RLN3 antibody and a polyclonal guinea-pig Syn antisera (#101004, Synaptic Systems; Göttingen, Germany). An average of three rats per group (P15, P17, P20 and adult) was used. For each rat, three sections from multiple rostrocaudal levels were collected covering the entire medial septal region (0.70 to 0.20 mm, relative to Bregma).

Sections were rinsed three times in 0.01 M PBS and incubated in blocking solution for 1 h at room temperature. Sections were then transferred to primary antibody solution containing 1:5 mouse anti-RLN3 and 1:500 anti-Syn overnight at room temperature. For RLN3 visualization, sections were rinsed twice in PBS and incubated in 1:400 goat anti-mouse-Alexa488 antibody (#115–545-146, Jackson Immunoresearch; Cambridge, UK) for 2 h at room temperature. For Syn visualization, sections were rinsed three times in PBS and transferred to a solution containing 1:400 goat anti-guinea pig-Cy3 antibody (#106–167-008, Jackson ImmunoResearch; Cambridge, UK) for 2 h at room temperature. Finally, sections were rinsed in PBS, mounted on clean slides and coverslipped using Fluorsave™.

### 2.8. Immunofluorescence triple-labeling for BDA, serotonin and RLN3

The relative localization of in the NI region of RLN3, serotonin and retrogradely-transported BDA in the NI region after MS injection was assessed by immunofluorescent detection of BDA – 5HT – RLN3. Briefly, free-floating brain sections were rinsed and incubated in blocking solution for 1 h and then transferred to primary antibody solution containing 1:2000 polyclonal rabbit anti-serotonin (S5545, Merck (Sigma-Aldrich); Madrid, Spain) and 1:5 mouse anti-RLN3 overnight at room temperature. For BDA – 5HT – RLN3 visualization, brain samples were incubated with 1:200 Alexa 488 donkey anti rabbit (#711–545-152, Jackson ImmunoResearch; Cambridge, United Kingdom), 1:200 Alexa 647 donkey anti-mouse (#715–605-150, Jackson ImmunoResearch; Cambridge, United Kingdom) and 1:200 Cy3 streptavidin (#016–160-084, Jackson ImmunoResearch; Cambridge, United Kingdom) for 2 h at room temperature. Finally, sections were mounted on clean slides and coverslipped using Fluorsave™.

### 2.9. Image acquisition and analysis

Immunofluorescence images were captured with a laser confocal scan unit (Leica DMi8, Leica Microsystems CMS GmbH, Wetzlar, Germany) equipped with argon and helio-neon laser beams attached to a DMIRB inverted microscope (Leica Microsystems). To avoid any bias, gain, offset and pinhole parameters were kept constant for all images.

The quantitative analysis of RLN3 fibers was performed using 5 μm images acquired from each MS section. Subsequently, using Image J software, the percentage of total area occupied by RLN3 fibers was quantified. The total area analyzed was optimized for each postnatal period analyzed, in order to eliminate any bias derived from the age-dependent size of the brain.

The level of RLN3 and synaptophysin coexistence was analyzed using 40× captured images of the MS. A total depth of 10 μm in each section was reconstructed with the Leica application suite (LASX, Leica software). The density of synaptophysin boutons along the length of RLN3 fibers was quantified.

Finally, each [BDA – 5HT – RLN3] image analyzed was comprised of a stack of 20 images, taken at 1024×1024 resolution with 20x lens, and was pre-processed by applying a maximal projection process using the Leica Application Suite LAS X (Leica Microsystems S.L.U; L’Hospitalet de Llobregat, Spain). BDA infusion site images comprised multiple images collected with a 5× objective using a tile scan option in the Leica Application Suite LAS X.

### 2.10. Statistical analysis

Possible differences in the level of septal RXFP3 mRNA at different postnatal ages were analyzed by a one-factor analysis of variance (ANOVA). A Shapiro–Wilk normality test was applied, and a one-way ANOVA was used to determine significant differences between groups at a probability less than 0.05 (*p* < 0.05) followed by a least significant difference (LSD) test between groups.

All analyses were conducted by an observer blinded to the experimental conditions. Both the influence of age and area in either percentage of RLN3-positive fibers or colocalized puncta were analyzed by a two-way ANOVA. Differences between ages or areas were estimated using a one-way ANOVA analysis. Statistical analyses for significant differences between groups employed the least significant difference (LSD) test. Probability was set as α < 0.05. GraphPad Prism V8 software (GraphPad, La Jolla, CA, USA) and Jeffreys’ Amazing Statistical Package (JASP, https://jasp-stats.org/) were used to perform the statistical analysis and graphical representation of the data.

All quantitative variables were represented as boxplots to display the distribution of data based on mean and minimum and maximum values, and were illustrated as violin plots to depict summary statistics and the density of each variable (Jeffreys’ Amazing Statistical Package, https://jasp-stats.org/).

## 3. Results

### 3.1. RXFP3 Expression and RLN3 distribution in the septal area of postnatal rats

**Figure 1:**
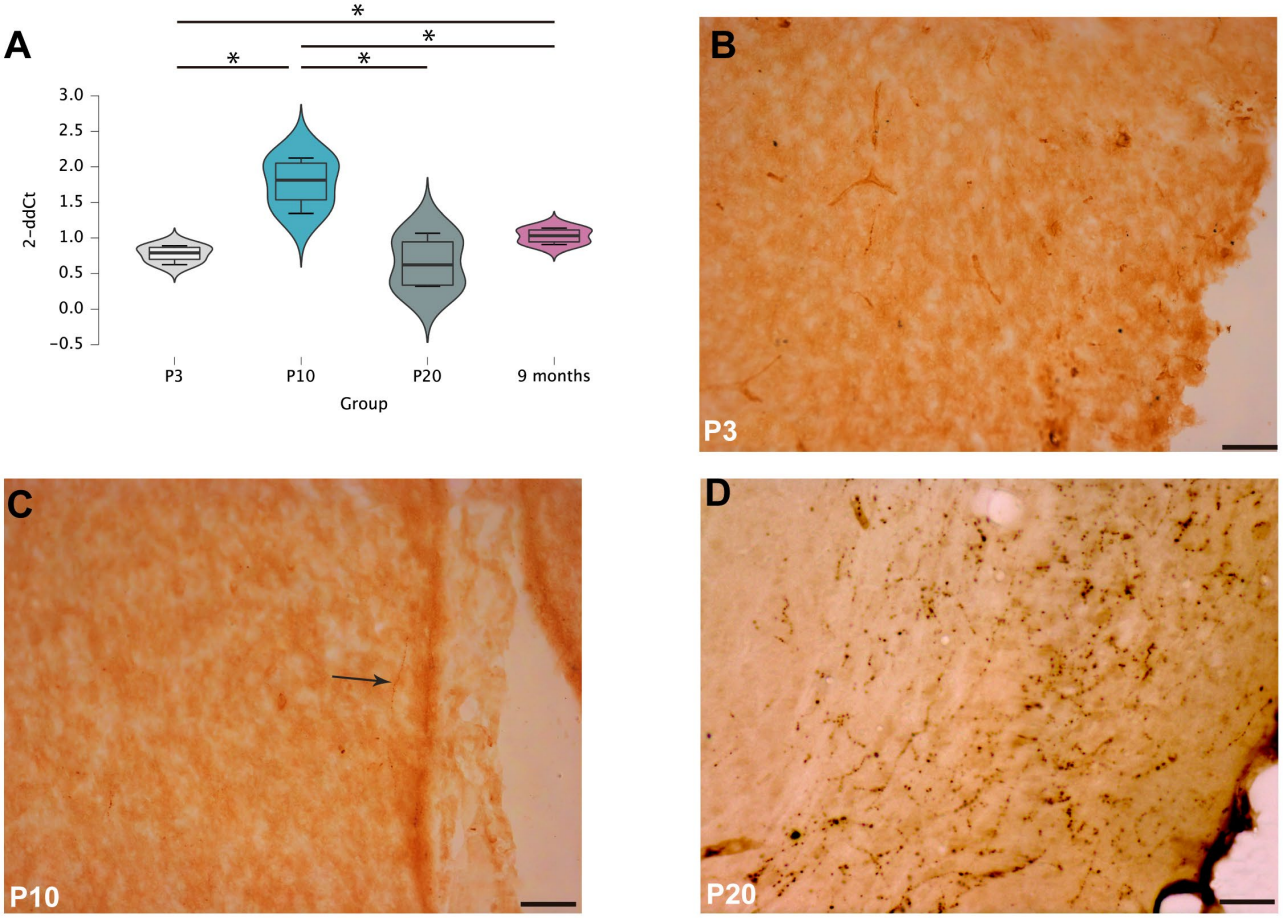
RXFP3 expression and RLN3 distribution in the septal area of postnatal rats **(A)**. RT-PCR analysis of RXFP3 mRNA levels in the septal area of P3, P10, P20 and 9 month-old rats revealed a peak at P10 and a normalization to adult concentrations by P20. Data are presented as mean ± SEM and were analyzed using one-way ANOVA followed by the LSD-text (*p < 0.05, **p < 0.01, ***p < 0.005) **(B–D)**. Immunohistochemistry for RLN3 revealed a few fibers at P3 **(B)**, dispersed RLN3 labeling in the septal area of P10 rats **(C)**, and dense RLN3 fibers in the horizontal diagonal band at P20 **(D)**. In **(C)**, the arrow highlights a RLN3 fiber. Calibration bar, 50 μm.

RNA extracted from the septal area of rats sacrificed at different postnatal ages (P3, P10, P20 and 9 months) was analyzed to determine the profile of RXFP3 expression during postnatal development (Figure 1A). One-way ANOVA analysis of RXFP3 mRNA levels revealed significant differences (*F* (3,12) = 13.016, *p* < 0.001) between age groups, with a significant increase in RXFP3 transcripts between P3 and P10 (*p* = 0.08, LSD test), whereas RXFP3 mRNA levels were decreased by P20 (*p* = 0.006) and were maintained at these levels in the adult rat brain (*p* = 0.02) (Figure 1A). In line with this RXFP3 expression profile, immunohistochemical detection of RLN3 fibers at three postnatal ages (P3, P10 and P20), revealed that RLN3 fibers appeared within the P10–20 window (Figures 1B–D).

### 3.2. Quantification of RLN3-positive fibers in the septum

**Figure 2:**
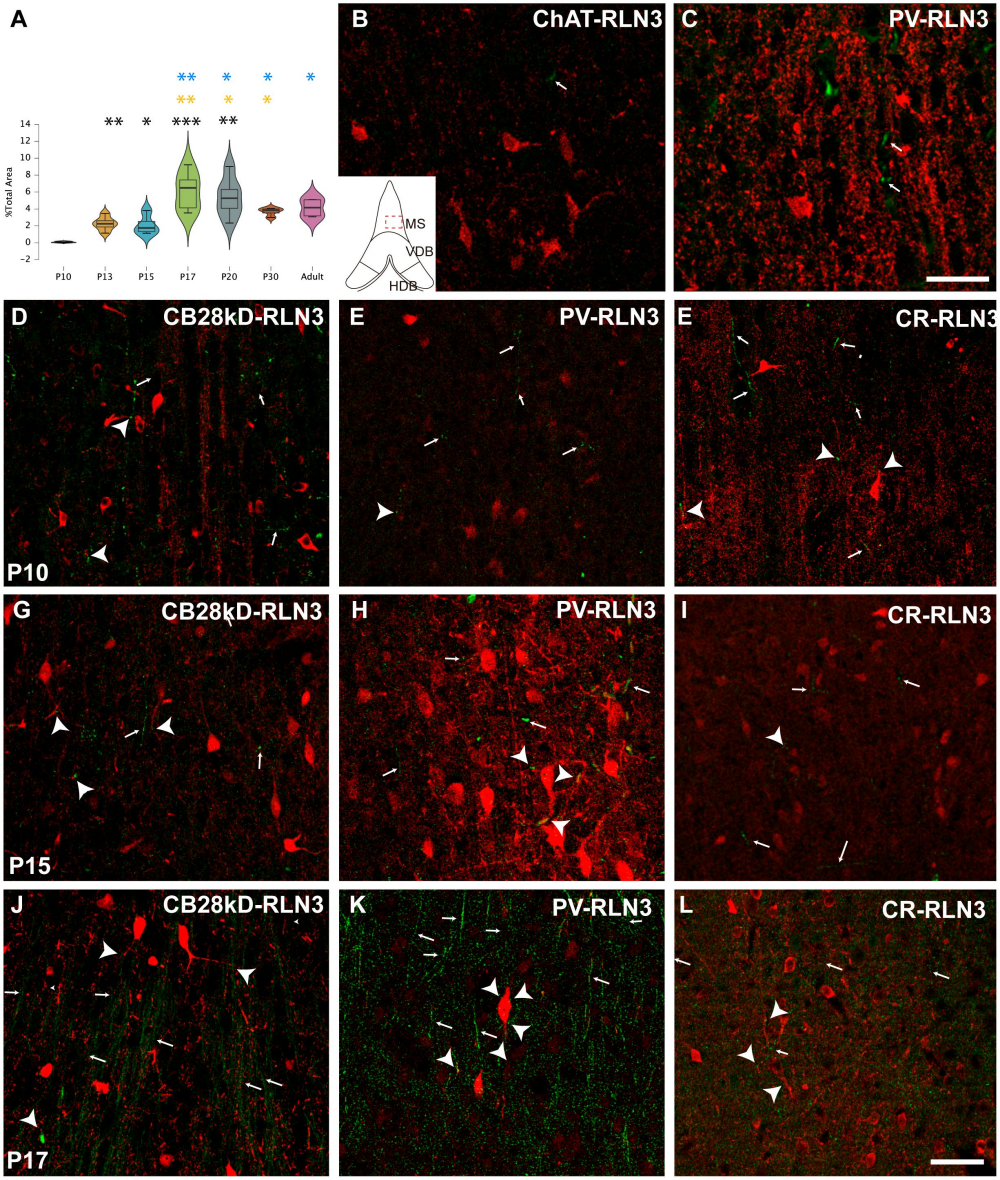
Development of the RLN3 innervation of the MS0 **(A)**. Violin plots of RLN3 fiber density during postnatal development from P10 to P20 and in adult rats **(B–L)**. Maximal projections of a total stack of 5 μm images displaying double-labeling of calcium-binding proteins or ChAT (red) and RLN3 (green) **(B,C)**. Double-labeling at P3 for ChAT and RLN3 **(B)**, and PV and RLN3 **(C)**. **(D–F)** double-labeling at P10 for CB28kD + RLN3 (**D**), PV + RLN3 (**E**) and CR + RLN3 (**F**). **(G–I)** double-labeling at P15 for CB28kD + RLN3 (**G**), PV + RLN3 (**H**) and CR + RLN3 (**I**). **(J–L)** double-labeling at P17 for CB28kD + RLN3 **(J)**, PV + RLN3 **(K)** and CR + RLN3 (**L**). An increased density in RLN3 fibers occurred between P15 **(G–I)** and P17 (**J–L**). Arrows point at RLN3 fibers, arrowheads point at putative contacts because of a close apposition between a positive RLN3 fiber and an IF structure. Calibration bar **(B–L)**, 50 μm. Colored symbols in bars represent trends and statistically significant differences among groups after *post hoc* analysis: *p < 0.05, **p < 0.01, ***p < 0.001.

**Figure 3:**
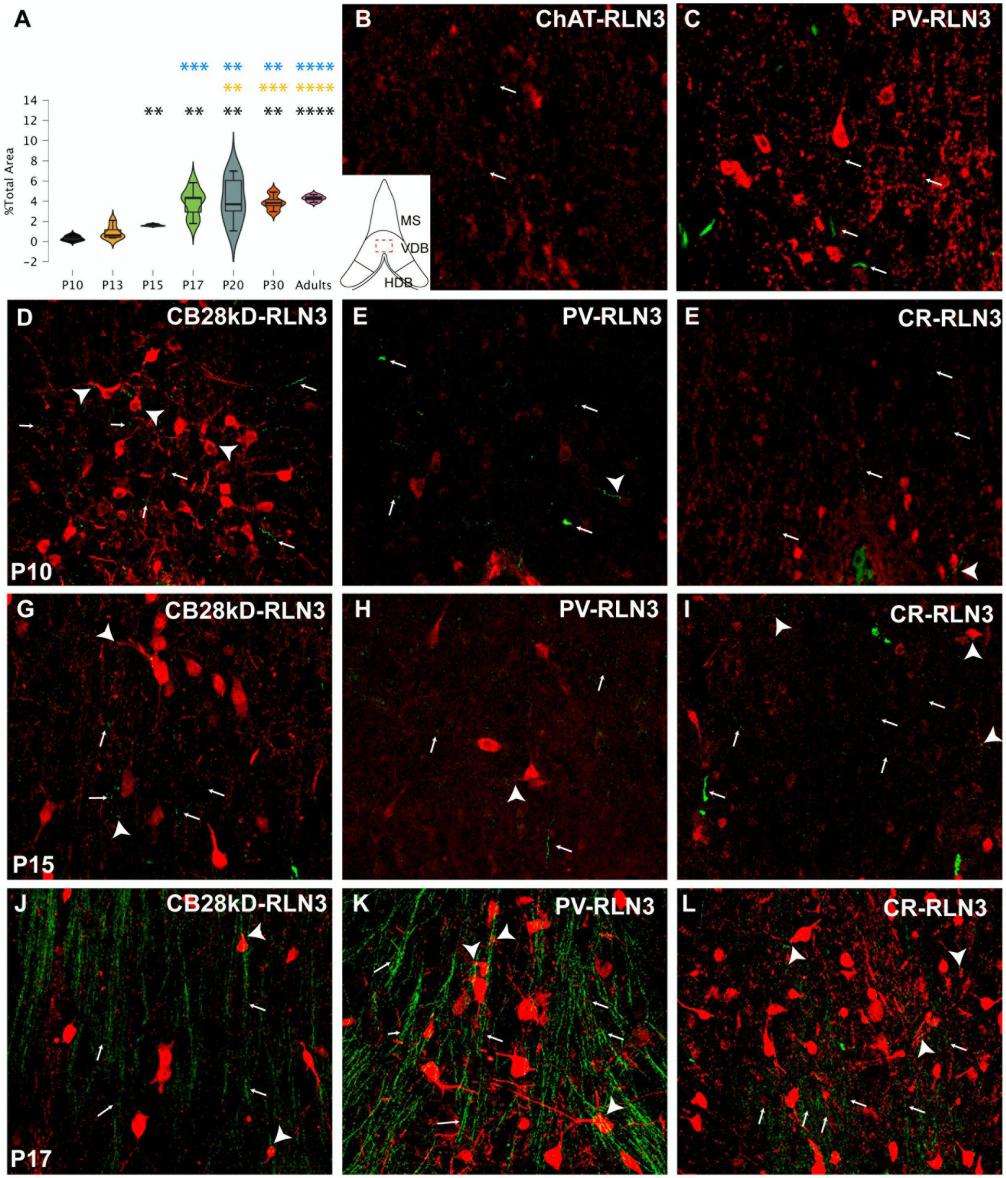
Development of the RLN3 innervation of the VDB **(A)**. Violin plots of the RLN3 fiber density during postnatal development from P10 to P20 and in adult rats **(B–L)**. Maximal projections of a total stack of 5 μm images displaying double-labeling of calcium binding proteins or ChAT (red) and RLN3 (green) **(B,C)**. Double-labeling at P3 for ChAT + RLN3 **(B)** or PV + RLN3 **(C)**. **(D–F)** double-labeling at P10 for CB28kD + RLN3 **(D)**, PV + RLN3 **(E)** and CR + RLN3 **(F)**. **(G–I)** double-labeling at P15 for CB28kD + RLN3 **(G)**, PV + RLN3 **(H)** and CR + RLN3 **(I)**. **(J–L)** double-labeling at P17 for CB28kD + RLN3 **(J)**, PV + RLN3 **(K)** and CR + RLN3 **(L)**. An increased density of RLN3 fibers occurred between P15 **(G–I)** and P17 **(J–L)**. Arrows point at RLN3 fibers, arrowheads point at putative contacts because of a close apposition between a positive RLN3 fiber and an IF structure. Calibration bar B-L, 50 μm. Colored symbols in bars represent trends and statistically significant differences among groups after *post hoc* analysis: *p < 0.05, **p < 0.01, ***p < 0.001, ****p < 0.0001.

**Figure 4:**
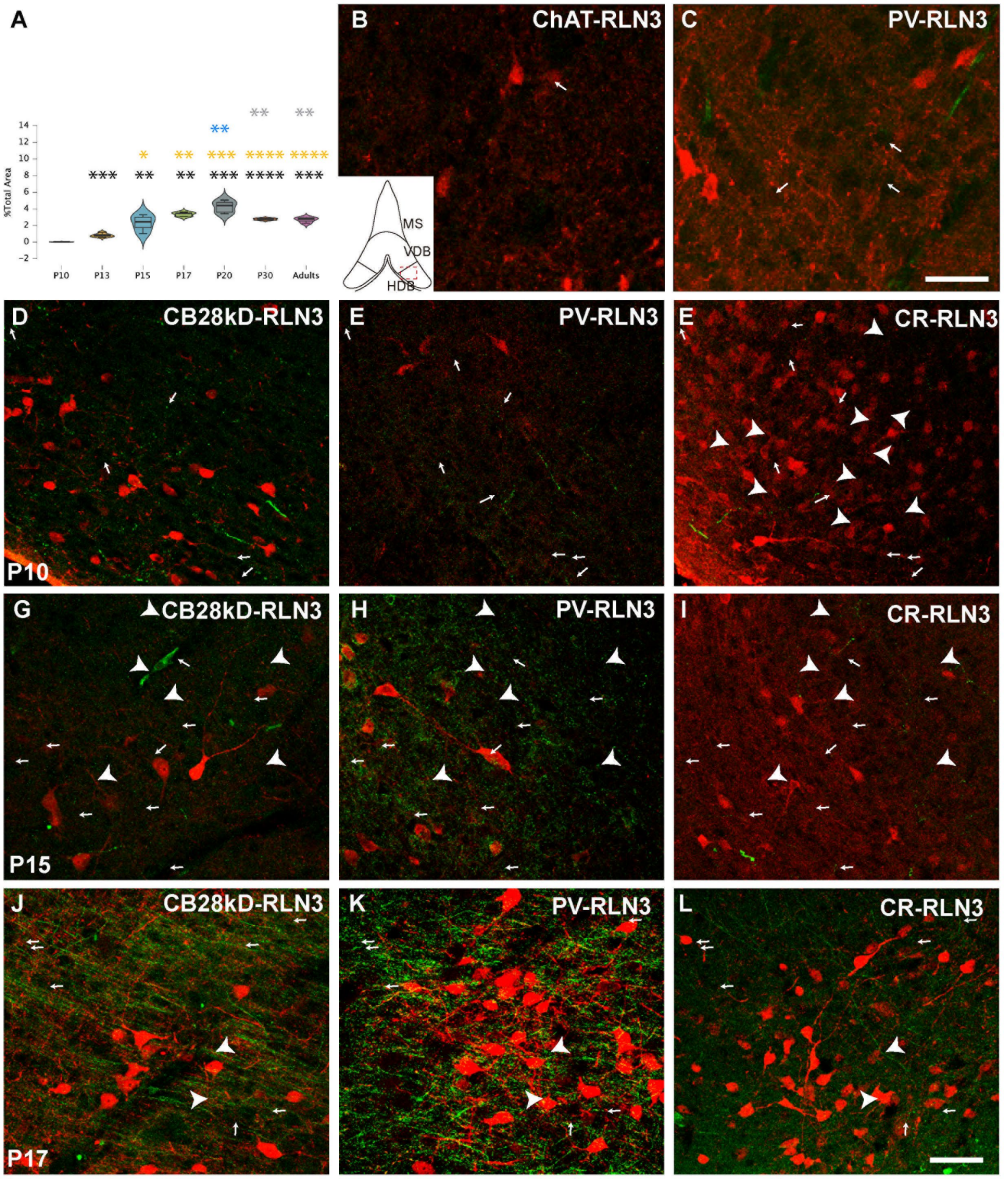
Development of the RLN3 innervation of the HDB **(A)**. Violin plots of the RLN3 fiber density during postnatal development from P10 to P20 and in adult rats **(B–L)**. Maximal projection of a total stack of 5 μm images displaying double-labeling of calcium-binding proteins or ChAT (red) and RLN3 (green) **(B,C)**. Double-labeling at P3 for ChAT + RLN3 **(B)** or PV + RLN3 **(C)**. **(D-F)** double-labeling at P10 for CB28kD + RLN3 **(D)**, PV + RLN3 **(E)** and CR + RLN3 **(F)**. **(G–I)** double-labeling at P15 for CB28kD + RLN3 **(G)**, PV + RLN3 **(H)** and CR + RLN3 **(I)**. **(J–L)** double-labeling at P17 for CB28kD + RLN3 **(J)**, PV + RLN3 **(K)** and CR + RLN3 **(L)**. Arrows point at RLN3 fibers, arrowheads point at putative contacts because of a close apposition between a positive RLN3 fiber and an IF structure. An increased density of RLN3 fibers occurred between P15 **(G–I)** and P17 **(J–L)**. Calibration bar **(B–L)**, 50 μm. Colored symbols in bars represent trends and statistically significant differences among groups after *post hoc* analysis: *p < 0.05, **p < 0.01, ***p < 0.001, ****p < 0.0001.

Five postnatal age groups (P10, P13, P15, P17 and P20) were studied and an average of three septal sections per rat and six rats per group were analyzed. The density of RLN3 fibers in MS, VDB and HDB was evaluated in each section. This density was calculated as the percentage of RLN3-positive fibers per total area (Figures 2–4). Two-way ANOVA analysis revealed that both area (*p* = 0.001) and group (p < 0.001) significantly influenced the percentage of RLN3-positive fibers. However, the mixed effect of both group and area did not influence this percentage (*F* (12,89) = 1.42, *p* > 0.05; two-way ANOVA).

Analysis of the MS revealed significant differences between groups (*F* (6 31) = 12, *p* < 0.0001; ANOVA). A more detailed analysis detected a significant difference between P10 vs. P13 (*p* = 0.001), vs. P15 (*p* = 0.02), vs. P17 (*p* = 0.0002), vs. P20 (*p* = 0.003), vs. P30 (*p* = 0.009) vs. adult (*p* = 0.01). Although P13 and P15 rats displayed the same density of RLN3-positive fibers, there was a significant increase at P17. Thus, from P17 onwards there were significant differences between both P13 (P17 vs. P13, *p* = 0.004; P20 vs. P13, *p* = 0.04 and adult vs. P13, *p* = 0.02) and P15 (P17 vs. P15, *p* = 0.007; P20 vs. P15, *p* = 0.04; P30 vs. P15, *p* = 0.02; and adult vs. P15, *p* = 0.02). Notably, between P17 and P20 there was no significant difference. In addition, there was a numerical decrease in fiber density at P30 compared to P17 and P20, but it was not significant. Therefore, there is an increase in the presence of RLN3 in the MS between P15 and P17, and this relative density is maintained through to adulthood (Figure 2A). The progressive increase in RLN3 immunofluorescence was accompanied by a progressive complexity in the types of neurons identified in the MS. Thus, at P3, scattered RLN3 fibers were observed in the MS, and at the same time, ChAT-positive neurons displayed some primary processes (Figure 2B), but neurons expressing calretinin and other calcium-binding proteins were small in size and poorly ramified (Figure 2C). At P10, neurons expressing CB28kD (Figure 2D), PV (Figure 2E) and CR (Figure 2F), displayed additional structural complexity and RLN3 fibers formed plexuses around these neurons and contacted neural processes. At P15, the RLN3 fiber density was not markedly different (Figures 2G–I), but PV neurons had gained dendritic complexity (Figure 2H). Finally, by P17 an increased density of RLN3 fibers was observed running in a ventrodorsal direction (Figures 2J–L).

In the VDB, significant differences in the RLN3 fiber density were detected between groups (*F* (6, 31) = 12.65, *p* < 0.0001; ANOVA;). Further analysis revealed a significant increase in RLN3 fiber density from P15 onwards, being higher than at P10 (*p* = 0.05) but not higher than P13 levels. The P10 VDB had a lower fiber density than P17 (*p* = 0.002), P20 (*p* = 0.002), P30 (*p* = 0.002) and adult (*p* < 0.0001) values. So apart from the change between P10 and P15, the most relevant increase in the density of RLN3-positive fibers occurred between P15 and P17. There was a significant increase compared to the younger age groups as P10 (*p* = 0.002), P13 (*p* = 0.0002) and P15 (*p* = 0.0008). In the older age groups (P20, P30 and adult), there was a significant increase in VDB RLN3 fibers compared to P17 levels, but not between these groups. Therefore, in the VDB a significant increase in RLN3 fibers was observed by P17, which was retained through to adulthood (Figure 3A).

Similar to observations in the MS, we observed a progressive increase in the complexity of neurons expressing CBPs in VDB that was parallel to the appearance of RLN3 fibers occurring by P17. A few dispersed RLN3 fibers were observed at P3 adjacent to ChAT (Figure 3B) and poorly developed PV neurons (Figure 3C). Substantially more RLN3 fibers were observed at P10, and in parallel, neurons containing CB28kD (Figure 3D), PV (Figure 3E) and CR (Figure 3F) displayed some increased complexity. Between P10 and P15 CBP-positive neurons displayed similar dendritic trees and no marked changes occurred in RLN3 fiber density (Figures 3G–I), while a marked increase in RLN3 fiber density occurred between P15 and P17 (Figures 3J–L). A change in the complexity of PV neurons was observed between P15 (Figure 3K) and P17 (Figure 3L).

Finally, in the HDB, significant differences were obtained between groups (*F* (6, 29) = 23.28, *p* < 0.0001; ANOVA). A significant increase in the density of RLN3 fibers was observed from P10 to P13 (*p* = 0.0002) and from P13 to P15 (*p* = 0.0002), and these levels of RLN3-positive fibers were maintained until adulthood, with no significant differences between the older age groups (P15, P17, P20, P30 and adult), although a peak density of RLN3 fibers was observed at P20, which was significantly higher than levels at P30 (*p* = 0.005) and in adult rats (*p* = 0.005) (Figures 4A–C).

The progressive increase in the complexity of neurons expressing CBPs occurred in parallel to the sudden appearance and spread of RLN3-positive fibers in the HDB at P17. A few dispersed fibers were observed at P3 between poorly arborized ChAT (Figure 4C) and PV neurons (Figure 4D). More RLN3 fibers were observed at P10 and at the same time, neurons containing CB28kD (Figure 4D), PV (Figure 4E) and CR (Figure 4F) displayed some primary dendritic trees. No morphological changes were observed in dendritic trees or density of RLN3 fibers between P10 and P15 (Figures 4G–I) and the most marked changes occurred between P15 and P17 particularly in the density of RLN3 fibers which ran parallel to the HDB surface forming dense plexuses (Figures 4J–L), with a corresponding complexity of PV neurons (Figure 3L).

### 3.3. Co-localization of RLN3 and synaptophysin immunofluorescence in the septum

Synaptophysin (Syn) and RLN3 immunofluorescence co-localization was analyzed to identify the presence of synaptophysin/RLN3-positive synapses/terminals on septal neurons. These studies revealed granular Syn-labeling around septal neuronal soma. Syn-positive boutons were clearly observed within the first 5–10 μm below the surface of coronal septal sections.

**Figure 5:**
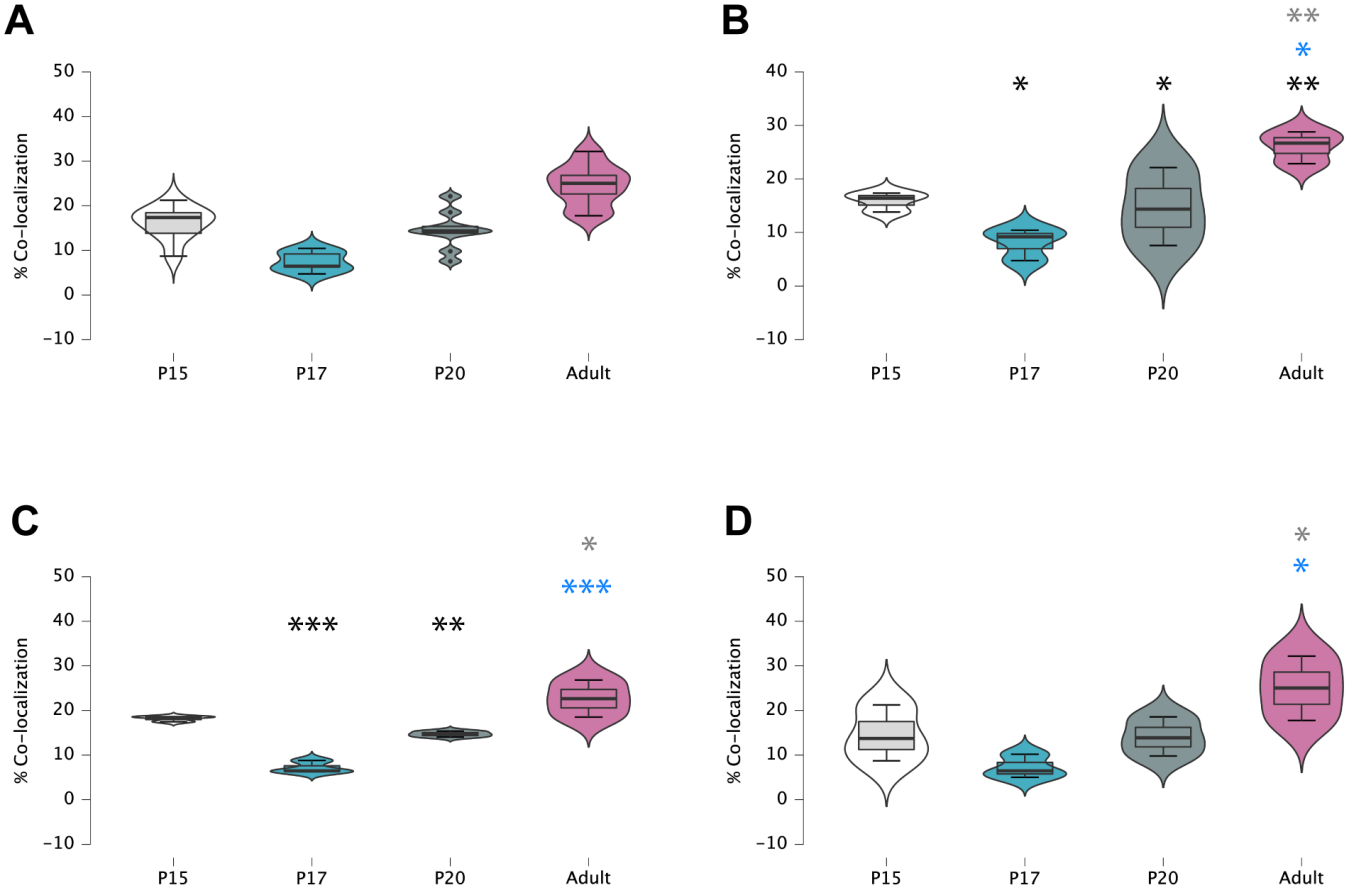
Violin plots of comparative mean/median values and percentiles of double-labeled RLN3/Syn vs. RLN3 puncta alone **(A)**, in MS **(B)**, VDB **(C)** and HDB **(D)**. Colored symbols in bars represent trends and statistically significant differences among groups after *post hoc* analysis: *p < 0.05, **p < 0.01, ***p < 0.001.

A two-way ANOVA was conducted that examined the effect of age and area on the level of co-localization observed. There was no significant interaction between the effects of age and area on co-localization (*F* (6, 24) = 0.335, *p* > 0.05). However, there was a significant effect of age on the level of synaptophysin/RLN3 co-localization (*p* < 0.001) (Figure 5A).

**Figure 6:**
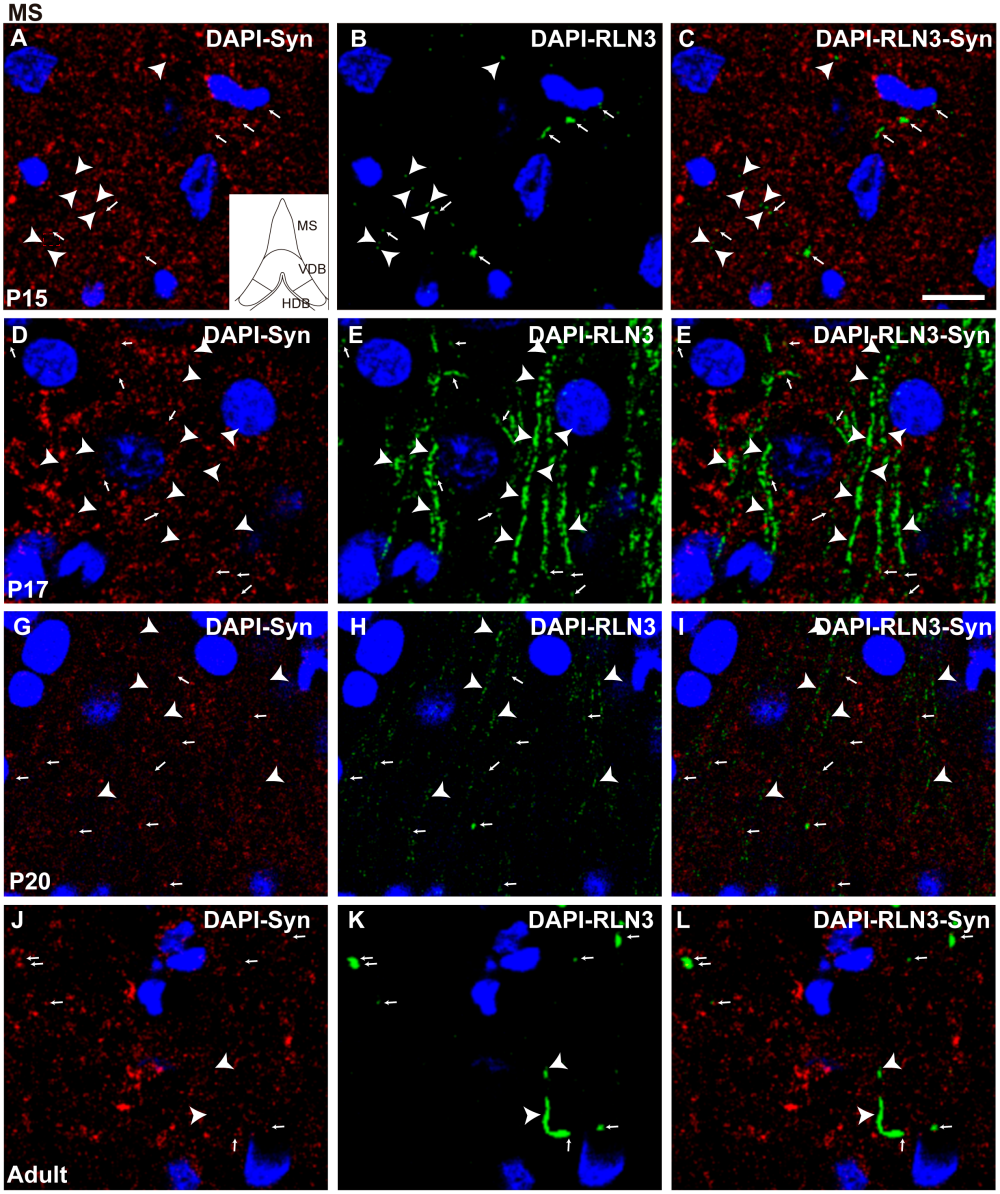
Single 0.5 μm confocal images of RLN3 and Syn immunofluorescence in the MS at different postnatal ages – P15 **(A–C)**, P17 (**D–F**), P20 (**G–I**), and adult **(J–L)** of the MS. Small arrows indicate double-labeled RLN3-Syn puncta, and arrowheads indicate RLN3 only fibers. Calibration bar, 10 μm.

At the MS level, there were significant differences between groups (*F* (3, 8) = 36.04, *p* < 0.0001; ANOVA). Further analysis revealed a significant decrease in the percentage of puncta co-localization between P15 and P17 (*p* = 0.019) and P15 and P20 (*p* = 0.03), but the level of co-localization was increased in adult MS, and was significantly higher than at P20 (*p* = 0.012), P17 (*p* = 0.002), and P15 (*p* = 0.007) (Figure 5B). Thus, two interesting patterns of puncta co-localization were observed at the MS level, an initial decrease between P15 (Figures 6A–C) and P17 (Figures 6D–F), which was maintained at P20 (Figures 6G–I), and thereafter an increase in the percentage co-localization in adult MS, which exceeded the levels detected at P15 (Figures 6J–L).

**Figure 7:**
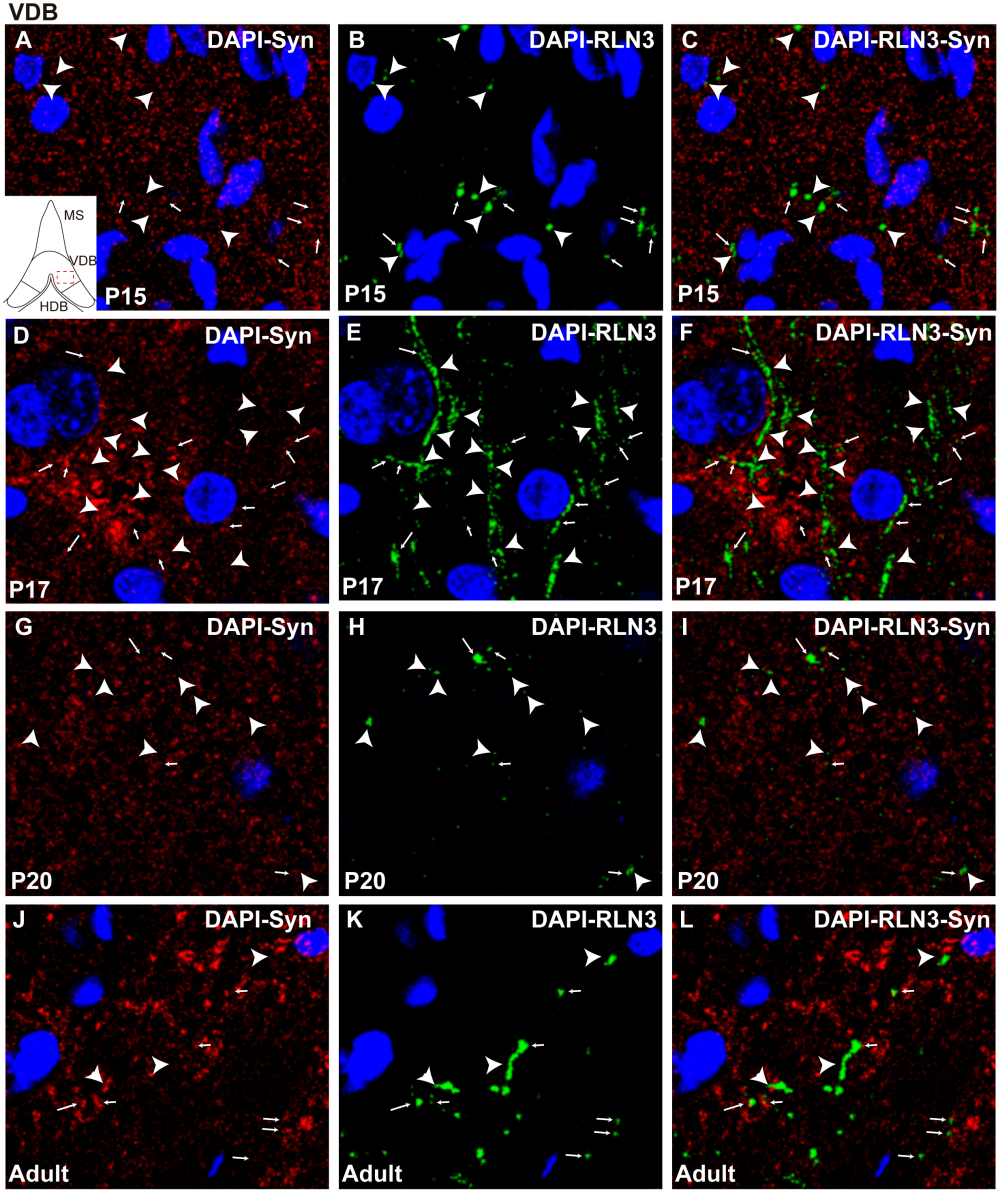
Single 0.5 μm confocal images of the VDB immunolabeled for RLN3 and Syn at different postnatal ages – P15 **(A–C)**, P17 **(D–F)**, P20 **(G–I)** and adult **(J–L)**. Small arrows indicate double-labeled RLN3-Syn puncta, and the arrowhead indicates RLN3 only fibers. Calibration bar, 10 μm.

In the VDB, there were also significant differences between groups (F (3, 8) = 22.1, *p* = 0.0003; ANOVA). A significant decrease in the percentage of puncta co-localization was observed between P15 and P17 (*p* = 0.0002), which was maintained at P20 (*p* = 0.009), while levels of co-localization had reverted to P15 levels in the VDB of adult rats. The adult VDB displayed a significant increase in the percentage of puncta co-localization compared to P17 (*p* = 0.0006) and P20 (*p* = 0.01) levels (Figure 5C). In summary, a decrease in the percentage of puncta co-localization was observed between P15 (Figures 7A–C) and P17 (Figures 7D–F), and thereafter an increase occurred from P17 to P20 (Figures 7G–I) that was maintained at adulthood (Figures 7J–L).

**Figure 8:**
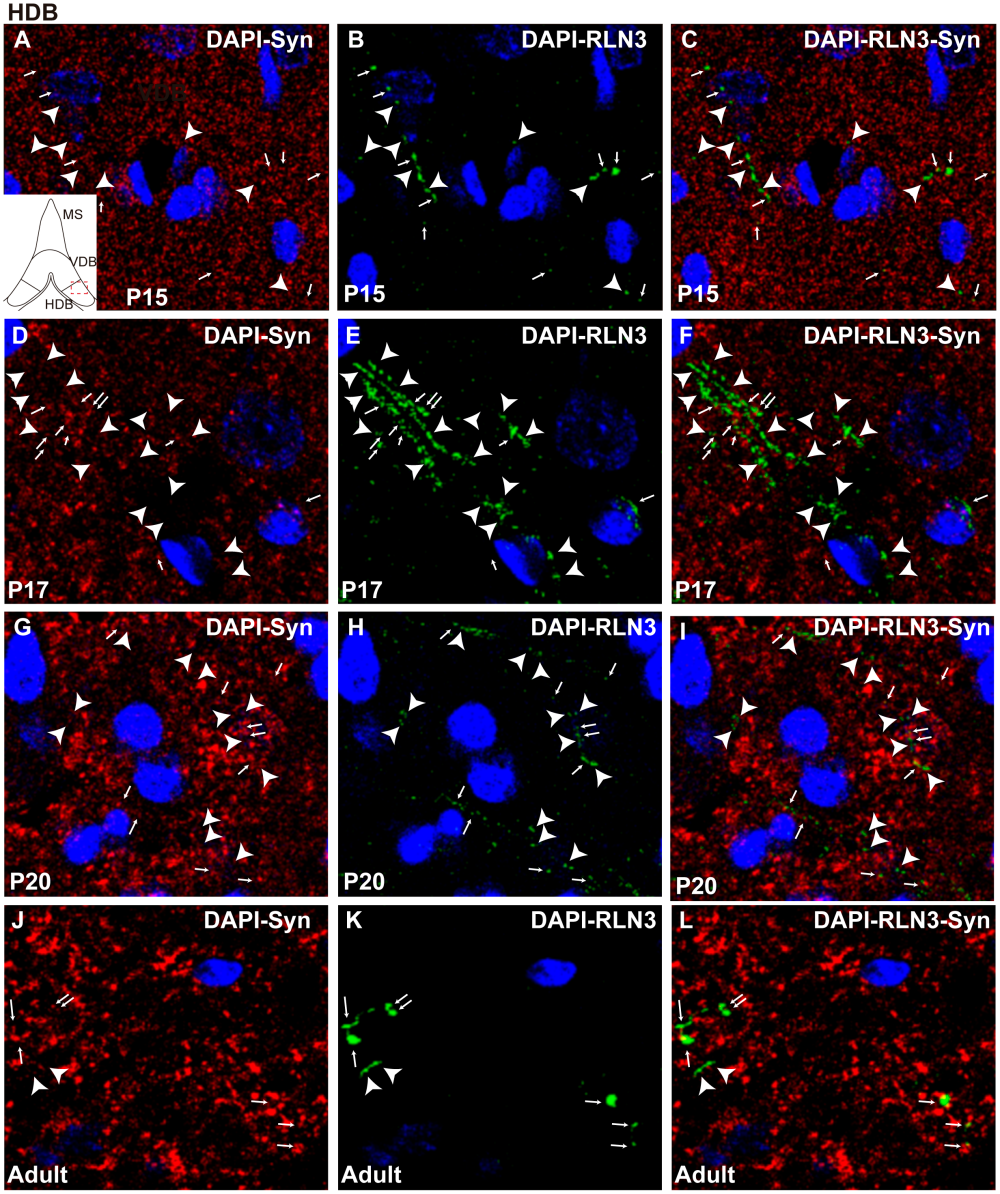
Single 0.5 μm confocal images of the HDB immunolabeled for RLN3 and Syn at different postnatal ages – P15 **(A–C)**, P17 **(D–F)**, P20 **(G–I)** and adult **(J–L)**. Small arrows indicate double-labeled RLN3-Syn puncta and the arrowhead indicates RLN3 only fibers. Calibration bar, 10 μm.

Finally, there were significant differences between groups in the HDB region (*F* (3, 8) = 6.97, *p* = 0.013; ANOVA) as observed in the other septal areas. However, in contrast to findings in the MS and VDB, no difference was observed in the percentage of RLN3-Syn puncta co-localization between P15 and P17, and levels remained similar at P20. However, a significant increase in co-localization was observed in adult HBD, compared to P17 (*p* = 0.016) and P20 (*p* = 0.03) (Figure 5D). Therefore, we postulate that there was a decrease in double-labeled fibers between P15 (Figures 8A–C) and P17 (Figures 8D–F) and a non-significant increase between P17 and P20 (Figures 8G–I), with a subsequent increase by adulthood (Figures 8J–L).

Generally, there was a decrease in the percentage of RLN3-Syn double-labeling throughout the septal area between P15 and P17, due to the infiltration of RLN3 fibers into the septal area that do not yet have their complete synaptic machinery. Thereafter, an increase in co-localization occurred due to the expression of synaptic proteins that contribute to the completion of this required machinery.

### 3.4. RLN3 Ni projections to medial septum

**Figure 9:**
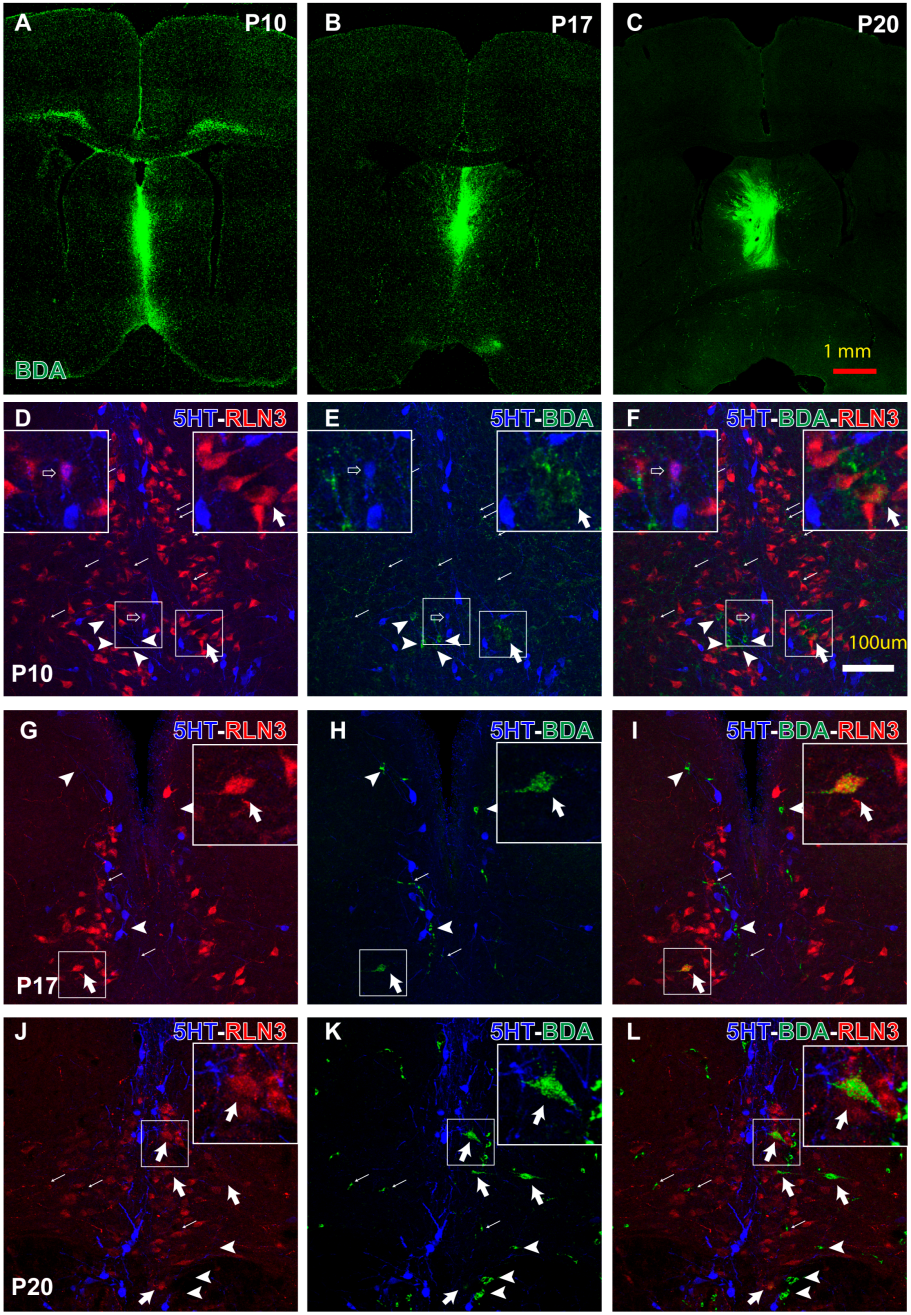
Anterograde and retrograde labeling in the pontine tegmental raphe/NI border at key postnatal ages, 3 days after prior BDA-3kD injections into the MS, combined with RLN3 and 5HT immunohistochemistry. **(A–C)** representative injection sites at P10 (survival P10–P13) **(A)**, P17 (survival P17–P20) and P20 (survival P20–P23) Calibration bar, 1 mm. **(D–F)** appearance of triple 5HT, RLN3 and BDA labeling in the NI between P10–P17. Inset, (left) is a magnified view of the boxed area (left), (illustrating a 5HT + RLN3 double-labeled neuron); inset (right) is a magnified view of the boxed area (right), illustrating a RLN3 + BDA double-labeled neuron. **(G–I)** appearance of 5HT + RLN3 + BDA triple-labeling in the NI between P17–P20. Inset is a magnified view of the boxed area, illustrating a RLN3 + BDA double-labeled neuron. **(J–L)** 5HT + RLN3 + BDA triple-labeling in coronal sections at the level of the NI after a BDA injection into the MS at P20 and processing at P23. Inset is a magnified view of the boxed area, illustrating a BDA + RLN3 double-labeled neuron. From **(D)** to **(L)**, arrows indicate anterogradely-labeled BDA fibers, arrowhead indicates single, BDA retrogradely-labeled neurons, open arrow indicates RLN3 + 5HT double + labeled neurons, and thick arrow indicates RLN3 + BDA double-labeled neurons. Calibration bar in **(L)** for **(D–L)**, 100 μm.

A neural tract-tracing experiment was conducted to assess the development of the RLN3 projection from the NI to the MS. Injections of low molecular weight (3kD) BDA were made into the MS at P10, P17 and P20. After 3 days, to allow for retrograde and anterograde transport of the tracer, rats were anesthetized and perfused at P13, P20 and P23, respectively, and processed for immunohistochemistry. Injection sites restricted to the septal area were obtained in three cases at each age (Figures 9A–C). RLN3, 5HT and BDA labeling was analyzed within the NI at each age.

Strong RLN3 immunofluorescence was observed in rats injected with BDA on P10 and largely did not overlap with 5HT immunofluorescence (Figures 9D–F). However, a few small cells were observed that appeared to be double-labeled for RLN3 + 5HT (insets, left; Figures 9D–F). Furthermore, a high density of BDA anterogradely-labeled fibers were observed surrounding and/or contacting RLN3- and 5HT-positive neurons. A few BDA-positive neurons were observed and some were RLN3-positive (insets, right; Figures 9D–F).

In rats injected with BDA at P17 and processed at P20, 5HT-positive neurons were aligned on both sides of the dorsal pontine midline at the caudal limit of the dorsal raphe nuclei and the rostral border of the NI (Figures 9G–I). A reduction in the density of anterogradely-labeled BDA fibers was observed in these sections. No RLN3 + 5HT double-labeled neurons were observed, but some neurons were RLN3 + BDA positive (insets, Figures 9G–I).

In coronal sections from rats injected at P20 and processed at P23, the border between 5HT-positive neurons in the dorsal and pontine raphe nuclei and RLN3-positive neurons in the NI was more evident (Figures 9J–L). There was no overlap between the RLN3 and 5HT neuron populations, and there was a visible reduction in the density of anterogradely-labeled BDA fibers in the NI. Concurrently, the number of BDA retrogradely-labeled neurons in the NI was increased and several of these neurons were RLN3-positive (insets, Figures 9J–L).

## 4. Discussion

These studies characterized the postnatal profile of RLN3-immunopositive nerve fibers in the rat medial septal complex, revealing the time of their first appearance between P15 and P17. The postnatal profile of septal expression of mRNA encoding RXFP3, the RLN3 receptor, was also examined using qRT-PCR and revealed expression at P3 through to adulthood, with an aged-related decline in the relative level of RXFP3 mRNA between P10 and P20, in line with an earlier report of the postnatal appearance of RXFP3 in rat whole brain (Miyamoto et al., 2008).

In parallel to RXFP3 expression and the developmental appearance of RLN3 fibers between P15 and P17, immunohistochemical detection of Syn and its relative level of co-localization with RLN3 indicate that ‘synaptic machinery’ within the ascending RLN3 inputs develops later than P17 and that the NI to MS projection becomes functional after the maturation of the innervation. In support of this idea, septal lesions made at P12 impaired eye blink conditioning on P21–23, but not on P17–19 (Harmon and Freeman, 2015). These results led the authors to postulate that the septohippocampal system and its subsequent control of hippocampal theta rhythm was active and able to facilitate eye blink conditioning between P19 and P21 (Harmon and Freeman, 2015). Others have highlighted the relevance of this time window in the ontogeny of hippocampal theta rhythm, as rhythmic activity of inhibitory postsynaptic currents at theta frequencies first appears between P16 and P25, and these currents are disrupted by the activation of presynaptic GABA_B_ receptors by the GABA_B_ receptor agonist, baclofen (Henderson and Jones, 2005).

The period between P10 and P20 is also critical for different forms of hippocampal- and septal-dependent learning and memory processes. For example, object recognition emerges at P17 in the rat (Krüger et al., 2012; Westbrook et al., 2014), the same age at which the acquisition and retention of object recognition memory in context develops (Ramsaran et al., 2016). In contrast, other forms of spatial learning, such as object location, become evident between P17 and P21 (Westbrook et al., 2014), and several types of contextual learning (i.e., context pre-exposure facilitation effect (Schiffino et al., 2011; Jablonski et al., 2012)) appear around this age. This period is when the RLN3-positive innervation invaded the medial septal complex. Although most forms of spatial and contextual learning are hippocampal-dependent (Mumby et al., 2002; Rudy et al., 2002; Barker and Warburton, 2011) some depend on the integrity of the septohippocampal connection (Cain et al., 2002; Easton et al., 2010; Dashniani et al., 2015). In this regard, we observed that interference with RLN3 signaling at the level of the medial septal complex resulted in disruption of the spontaneous alternation test in adult rats (Ma et al., 2009; Albert-Gasco et al., 2018), and permanent depletion of Rxfp3 mRNA/protein in the medial septum of a transgenic mouse line (i.e., a floxed RXFP3 mouse) resulted in a strong impairment of spatial memory in the Morris water maze (Haidar et al., 2019).

Neonatal pups (<P7 days) do not display hippocampal theta (Leinekugel et al., 2002), but hippocampal theta emerges very early during postnatal development (Leblanc and Bland, 1979). However, the ‘mismatch’ between the appearance of hippocampal theta and the RLN3 innervation of the medial septum could be due to the existence of different forms of hippocampal theta, including a form of theta that can be generated intrinsically in *in vitro* preparations and in *in vivo* preparations, which is septal-independent and septal-related, respectively (Jacob et al., 2017).

The RLN3 innervation of the septal complex appears largely between P15 and P17. Unlike this peptide system, the monoamine transmitter, serotonin is present at birth, but during subsequent weeks, serotoninergic fibers became thinner, varicose, and re-oriented until they adopt their final appearance by the end of the third postnatal week (Dinopoulos et al., 1997). We observed a maturation in the segregation between the caudal part of the dorsal and pontine raphe and the NI. It is possible that both processes are interconnected, as, for example, acute depletion of brain serotonin during development resulted in an increased activation of the transcription of RLN3 gene in the NI (Miyamoto et al., 2008).

Synaptophysin (Syn) is the most abundant integral membrane glycoprotein present in the synaptic vesicles of neurons (McMahon et al., 1996). Syn immunoreactivity has been commonly used as a presynaptic marker for the quantification of synapses (Calhoun et al., 1996). During adult life, Syn is not present at constant levels, in fact, variations in hippocampal Syn expression have been observed with aging and after physical exercise in rodents (Casoli et al., 1996; Xu et al., 2019). These alterations are reported to be associated with modifications of presynaptic inputs. We previously demonstrated that in the septal complex of adult rats, tight contacts occur between RLN3-positive terminals and other neuronal types, including neurons expressing calcium-binding proteins (Olucha-Bordonau et al., 2012). Accordingly, the current results indicate some synaptic remodeling between P15 and P17 in the medial septal complex, in accordance with synaptic remodeling observed in other areas, which has been linked to the establishment of new connection patterns (Castejón et al., 2004; Atkinson and Williams, 2009).

The septal area is targeted by ascending projections arising from the NI and RLN3 is involved in the modulation by the septum of theta rhythm generation (Olucha-Bordonau et al., 2012). The rat MS receives serotonergic projections from the raphe nuclei (Vertes et al., 1999) and RLN3-positive projections emerging from the NI (Olucha-Bordonau et al., 2003; Tanaka et al., 2005; Ma et al., 2007). Furthermore, the main source of the serotonergic innervation of the MS is the median raphe nucleus (Leranth et al., 1999), which not only receives projections from the NI (Goto et al., 2001; Olucha-Bordonau et al., 2003), but projects to the dorsal hippocampus (McKenna and Vertes, 2001). The results of the BDA (3kDA) labeling study demonstrated three characteristic profiles. While at early postnatal ages (P10–P13), a few cells double-labeled for 5HT + RLN3 were observed in the NI, at later ages the differentiation between the RLN3 and 5HT neuron populations was fully established. This differentiation began between P17–P20 and concluded between P20–P23. At the same time, a higher density of neurons with BDA anterograde-labeling observed at initial postnatal ages, decreased during the subsequent postnatal ages. Finally, the number of retrogradely-labeled neurons increased with postnatal age, and most of these neurons contained RLN3 immunoreactivity. Therefore, our results are in agreement with those in adult rats demonstrating a distinct pattern of RLN3 and 5HT neurons/fibers at the level of the NI and MS.

These results suggest that the final configuration of the rat septal complex, and especially the MS, emerges at the end of the third postnatal week, coinciding with the time that fibers expressing serotonin and RLN3 acquire their final conformation. These events correlate with the post-weaning period, during which pups begin to lead an autonomous life and contextual and declarative memories are required for independent living and survival (Workman et al., 2013). In this study, we identified the timing of the occurrence of RLN3 fibers in the MS and observed its relevant coincidence with pup weaning and the commencement of a more autonomous life.Therefore, future research should address issues related to the configuration of this system such as the signaling mechanisms directing RLN3 fibers to project to the MS and to establish synaptic relationships with their targets. Furthermore, it will be of interest to determine whether the arrival of the RLN3 fibers can regulate other developmental processes as suggested for the serotoninergic regulation of the RLN3 developmental profile (Miyamoto et al., 2008).

## Data availability statement

The raw data supporting the conclusions of this article will be made available by the authors, without undue reservation.

## Ethics statement

The animal study was reviewed and approved by Comité de Ètica i Experimentació Animal, Universitat Jaume I.

## Author contributions

FR-B conceived the work, planned the PCR and IF procedures, acquired the images, read, and corrected the successive versions, IG-M, performed the tracing experiments, contributed to image quantifications, and description of the tracing experiments, JL contributed to image acquisition and quantification, MN-S contributed to the analysis of tracing experiments, EC-G contributed to the analysis of multiple immunofluorescence and the analysis of data, AG read and corrected successive views of the manuscript, FO-B conceived the work planned the experiments, contributed to writing the first version of the manuscript and read and corrected successive versions of the manuscript. All authors contributed to the article and approved the submitted version.

## Funding

This research was funded by the Fundación Alicia Koplowitz, Spain, grant number 19I436 (FO-B, FR-B, EC-G); the Spanish Ministerio de Ciencia, Innovación y Universidades, grant number RTI2018-095698-B-I00 (FO-B, EC-G, FR-B, IG-M); AICO Generalitat Valenciana, grant number AICO/2021/246 (FO-B, FR-B, EC-G), the UJI Predoctoral Program PREDOC/2021/19 (MN-S), the UJI Postdoctoral Program of the UJI POSDOC/2021/19 (IG-M); and Universitat Jaume I, grant numbers UJI-A2017-17 (FR-B), UJI-B2019-54 (FO-B), and UJI-A2020-20 (EC-G).

## Conflict of interest

The authors declare that the research was conducted in the absence of any commercial or financial relationships that could be construed as a potential conflict of interest.

## Publisher’s note

All claims expressed in this article are solely those of the authors and do not necessarily represent those of their affiliated organizations, or those of the publisher, the editors and the reviewers. Any product that may be evaluated in this article, or claim that may be made by its manufacturer, is not guaranteed or endorsed by the publisher.
